# Service Evaluation of the Just Right State (JRS) Programme, Step 3 Child and Adolescent Mental Health Services (CAMHS), Child and Family Clinic, Belfast Trust, Northern Ireland (March-December 2020)

**DOI:** 10.1192/bjo.2022.390

**Published:** 2022-06-20

**Authors:** Catherine Gillanders, Katie Jamieson

**Affiliations:** 1 Young People's Centre Step 3 CAMHS, Belfast, United Kingdom; 2 ASD Service, Belfast, United Kingdom

## Abstract

**Aims:**

COVID-19 resulted in dramatic shifts in how interventions are provided within mental health services, creating the opportunity to virtually deliver JRS groups to parents of young people attending Belfast CAMHS. This is a sensory attachment intervention that facilitates the process of self-regulation and co-regulation through the use of food, sensory activities and an enriched environment provision. It is currently facilitated by CAMHS clinicians via video calls over 4 consecutive weeks.

1. Evaluate the effectiveness of the virtual JRS intervention
2. Measure discharge rates after JRS intervention to examine if attendance at JRS at the point of entry into CAMHS can lead to more timely discharge due to targeted early intervention
3. To capture parent and clinician feedback focusing on the challenges and improvements that have occurred due to this adapted delivery of services

**Methods:**

A systematic database search was conducted examining number of parents who have attended overall; weekly attendance; Did Not Attend rate; length of time between CAMHS initial assessment and JRS intervention; number of families discharged after JRS and number of families allocated to partnership/medic after JRS.

CAMHS clinicians (not directly involved in facilitating JRS intervention) gathered qualitative feedback from families (via phone calls with parents who provided consent).

**Results:**

132 parents were invited between March-December 2020. 41 families have been discharged, 60 families have been allocated to partnership or medic and 31 are awaiting future JRS groups due to non-engagement, or a further review by JRS facilitators or a CAMHS clinician that they are already allocated to.

Five parents provided positive qualitative feedback.

**Conclusion:**

As JRS has engaged a high number of parents in a relatively short time -period, it would be helpful to further explore its effectiveness as a first line intervention in CAMHS, thereby informing service delivery moving forward.

